# The relationship between burden of childhood disease and foreign aid for child health

**DOI:** 10.1186/s12913-017-2540-5

**Published:** 2017-09-15

**Authors:** J. Clay Bavinger, Paul Wise, Eran Bendavid

**Affiliations:** 1 University of Michigan Medical School, Ann Arbor, MI USA; 2 Center for Health Policy and the Center for Primary Care and Outcomes Research, Stanford University, Stanford, CA USA; 3 The Division of General Medical Disciplines, Stanford University, Stanford, CA USA

## Abstract

**Background:**

We sought to examine the relationship between child specific health aid (CHA) and burden of disease. Based on existing evidence, we hypothesized that foreign aid for child health would not be proportional to burden of disease.

**Methods:**

In order to examine CHA and burden of disease, we obtained estimates of these parameters from established sources. Estimates of disability adjusted life years (DALYs) in children (0–5 years) were obtained from the World Health Organization for 2000 and 2012. The 10 most burdensome disease categories in each continent, excluding high-income countries, were identified for study. Descriptions of all foreign aid commitments between 1996 and 2009 were obtained from AidData, and an algorithm to designate the target diseases of the commitments was constructed. Data were examined in scatterplots for trends.

**Results:**

The most burdensome childhood diseases varied by continent. In all continents, newborn diseases, vaccine-preventable diseases (lower respiratory diseases, measles, meningitis, tetanus, and pertussis), and diarrheal diseases ranked within the four most burdensome diseases. Infectious diseases such as malaria, tuberculosis, and HIV were also among the ten most burdensome diseases in sub-Saharan Africa, and non-communicable diseases were associated with much of the burden in the other continents. CHA grew from $7.4 billion in 1996 to $17.7 billion in 2009 for our study diseases. Diarrheal diseases and malnutrition received the most CHA as well as the most CHA per DALY. CHA directed at HIV increased dramatically over our study period, from $227,000 in 1996 to $3.4 billion in 2008. Little aid was directed at injuries such as drowning, car accidents, and fires, as well as complex medical diseases such as leukemia and endocrine disorders.

**Conclusion:**

CHA has grown significantly over the last two decades. There is no clear relationship between CHA and burden of disease. This report provides a description of foreign aid for child health, and hopes to inform policy and decision-making regarding foreign aid.

**Electronic supplementary material:**

The online version of this article (doi:10.1186/s12913-017-2540-5) contains supplementary material, which is available to authorized users.

## Background

Financing of health goods and services in developing countries by donor nations and organizations has greatly increased in the last 25 years; health assistance increased from $6.9 billion in 1990 to $35.9 billion in 2014 [1]. Development assistance makes up an important share of healthcare resources in many countries. In low-income sub-Saharan African countries, health aid constituted 40% of total health expenditure in 2012 [1].

Multiple organizations track the sources and flows of health aid. The World Bank, the Organization for Economic Cooperation and Development (OECD), and the Institute for Health Metrics and Evaluation (IHME) all collect and synthesize data on health aid, and funding patterns have been the focus of multiple citations. This analysis focuses on child health aid (CHA), and our goal is to provide an accurate and complete depiction of CHA in relation to morbidity. We will describe a paucity of available evidence specific to CHA, and implore for more transparency in health aid and a more centralized databank, so that future research and policy decisions can be made using complete and detailed information. Previous research has highlighted the variation in childhood mortality across countries and continents, and understanding current CHA allocation is an important aspect of identifying and addressing current mortality trends [2]. We believe that our work will help better describe CHA distribution and will allow better assessment of strategies for optimal CHA distribution so that maximum impact may be achieved.

To date, “child health” has been treated as one (or part of a broader maternal, newborn, and child health) category of health aid, and positioned in comparison with other categories such as HIV/AIDS, tuberculosis, and malaria. The absence of detailed information on the specific components of CHA has prohibited a comparison of CHA patterns and disease burden among children. Our goal is to break down CHA into detailed categories in order to better understand how allocation patterns line up with disease burden.

Although there is little evidence specific to CHA, the evidence regarding overall foreign assistance disbursement patterns has found important trends. At the global level, health aid and disease burden are poorly aligned [3, 4]. Sridhar and Batniji found that funding per death varies widely between different diseases: $1029 was given per HIV/AIDS death, and only $3.21 per death for non-communicable diseases [4]. Denny and Emanuel write that the health assistance from the United States for diseases that claim an overwhelming number of child deaths, such as diarrheal diseases and respiratory infections, is significantly less than that targeted toward more publicized diseases such as HIV/AIDS, despite the fact that there are cost-effective treatments for diarrhea and respiratory infections [5].

We sought to explore the distribution of disease-specific CHA by continent, by utilizing a dataset with information on country-specific, disease-specific foreign assistance for health. We then examined the alignment of CHA with childhood country-specific burden of disease. In keeping with the evidence regarding overall foreign assistance for health, we hypothesized that CHA is not distributed proportional to burden of disease. We aim to inform policy and decision-making regarding CHA.

## Methods

We performed a cross sectional and longitudinal analysis to examine the associations between burden of disease and receipt of CHA in low- and middle-income countries worldwide from 1996 until 2009.

### Countries

We included only low- and middle-income countries, as defined by World Bank, for year 2000 [6]. Countries were grouped by continent according to World Bank (Additional file 1) [6]. Because our data source for burden of disease did not include countries with a population of less than 300,000 persons, such countries have been excluded from our analysis.

### Burden of disease

Estimates of burden of disease for children younger than 5 years were obtained from the World Health Organization (WHO), which provides disability-adjusted life years (DALY) organized by disease, age group, and country for years 2000 and 2012 [7]. Diseases for which similar technologies could be used were combined into disease categories, so that the search strategy could most accurately account for CHA. For example, vaccine-preventable diseases we grouped and included lower respiratory diseases, measles, meningitis, tetanus, and pertussis. Newborn diseases included preterm birth complications, birth asphyxia and trauma, neonatal sepsis, congenital heart anomalies, and neural tube defects. We included up to the ten most burdensome disease categories in each continent. We identified ten as a number which included all the major recipients of funding and also included the next several burdensome diseases, which in many cases received little to no funding. To accomplish this, we included the 20 most burdensome single diseases in each continent, and capped the number of categories at ten. As such, not all continents have ten categories, depending on how many categories the diseases were grouped into.

### Foreign aid

We utilized AidData research release 2.1, which is fully updated through 2009 [8]. This database includes extensive project-level information on all sectors of foreign donations, including health, education, and infrastructure. AidData includes all multilateral and bilateral donation sources, but does not include private donations or non-governmental organization (NGO) donations. As such, it includes the major sources of foreign aid, including bilateral aid from countries such as the United States and the UK, international groups such as the United Nations, large foundations including the Bill and Melinda Gates Foundation, and public-private partnerships including the Global Fund to Fight AIDS, Tuberculosis, and Malaria (GFATM) and GAVI. While these donors contribute a majority of foreign aid, it is important to recognize that other NGOs and private foundations are responsible for 15.8% of health aid from 1990 to 2014 [1], and are not included in this analysis.

Importantly, AidData includes a free-text description of the purpose of each foreign aid commitment, as well as the amount of aid and identity of donor and recipient. We developed an automated algorithm to search for words indicative of foreign aid for certain diseases. By running this algorithm on the AidData data, we identified foreign aid commitments targeted towards specific diseases. While AidData reports both disbursements and commitment amounts, previous research has described that disbursements are often under-reported and so other authors have used commitments or have tried to estimate actual disbursement from reported disbursement [9]. Because of this, we have chosen to analyze CHA according to commitment amounts.

To construct the automated algorithm, we used ICD-10 codes that the WHO associated with disease categories, so that categories of foreign aid matched closely with categories of burden of disease (Additional file 1). For instance, diarrhea is associated with ICD-10 codes A00-A09. From these, we developed a search tool that searched for words indicative of these ICD-10 codes, such as “diarrhea”, “giardia”, “rotavirus” and “norwalk”. Further, words indicative of treatments for diseases were included as well, such as “oral rehydration therapy” for diarrhea. After the search algorithm was run, we summed the amount of foreign aid commitment targeted to each disease, by country and by year. If foreign aid projects include words indicative of multiple diseases, the amount of the commitment was split evenly between the target diseases. Although the aid likely was not truly committed equally, we believe this is the most accurate method of dividing the commitment given that the actual allocation was not available.

Our search included key words for aid categories that are not always counted under health aid. Thus, in our search tool of aid for diarrhea, we included “drinking water,” which matters for reducing the burden of diarrheal illnesses, but is commonly excluded from estimates of health aid because it is not a medical intervention. Further, “food aid” was included in our search tool for aid targeted towards malnutrition, which, again, is commonly excluded in estimates of health aid. While lack of food and clean drinking water may be considered risk factors for disease rather than primary diseases, we do believe that these risk factors can be mapped accurately onto malnutrition and diarrheal diseases, and so are appropriate to include in our analysis.

Further, because this analysis is focused on childhood diseases and foreign aid for children, we needed to ensure that the aid we searched for was targeted towards children. Many diseases largely impact children, so any aid targeted to that disease is likely focused on child heath. Using the DALY data, we calculated the percent burden of a disease on children as a percentage of the entire population. For any disease in which greater than 30% of burden was in children, we included all foreign aid in our analysis, regardless of whether the description of the aid commitment specifically mentioned children. These included malaria, diarrhea, protein malnutrition, anemia, newborn diseases, vaccine-preventable disease, fire, and drowning. However, for diseases in which less than 30% of burden was in children, aid was only included in our analysis if the project description included words indicative of child health, such as “child”, “baby” and “infant.” These diseases were tuberculosis, HIV, syphilis, motor vehicle trauma, leukemia, and endocrine diseases. The distinction between diseases whose burden was or was not concentrated in children ensured that all CHA targeted towards diseases of children did not need to specify that a child health focus, while maintaining the child-specific nature of our analysis by requiring that health aid targeted at diseases not specific to children did include an indication of child health focus.

### Analysis

The search algorithm and data analysis were performed using STATA (StataCorp. 2009. Stata Statistical Software: Release 11. College Station, TX: StataCorp LP). The CHA received by each continent was calculated by year and by disease. CHA was also examined in relation to the corresponding amount of DALY, so that foreign aid relative to burden of disease could be examined. Data is presented in data table format and graphical scatterplot form to allow for visual inspection, both by region and with worldwide summary data. A logarithmic scale is used to better visualize the spread of child health aid. DALY were only available for years 2000 and 2012, so the estimates for 2000 were used as a surrogate for 1996 and the estimates for 2012 were used for 2009. For the scatterplot of DALY in relation to DALY, a narrow bandwidth (0.6) lowess curve is used to fit the trends.

## Results

Foreign aid for child health grew from $7.4 billion in 1996 to $17.7 billion in 2009 for our study diseases (Table 1). Diarrheal diseases and malnutrition received the most aid; diarrheal diseases were targeted with over $7 billion in 2009, and $4 billion was targeted at malnutrition in 2009. Aid directed at HIV increased dramatically over our study period, from $227,000 in 1996 to $3.18 billion in 2009. Comparatively little aid was directed at conditions of physical injury such as drowning ($1 million in 2009), car accidents (no CHA), and fires ($28 million) as well as complex medical diseases such as leukemia (no CHA) and endocrine disorders (no CHA).

**Table 1:** Worldwide Foreign aid for child health

Year	Total Donations	Anemia	Diarrhea	Drowning	Endocrine	Fire	HIV	Leukemia	Malaria	Malnutrition	Newborn	Road	Syphilis	TB	Vaccine
1996	$7440	$0	$6150	$0	$0	$0	$0	$0	$6	$1140	$83	$0	$0	$0	$52
1997	$7740	$0	$5780	$0	$0	$1	$2	$0	$24	$1210	$654	$0	$0	$0	$64
1998	$10,100	$0	$6120	$0	$0	$0	$4	$0	$8	$3380	$532	$0	$0	$0	$35
1999	$11,100	$0	$5400	$0	$0	$0	$118	$0	$13	$5020	$440	$0	$0	$1	$68
2000	$12,400	$0	$8340	$0	$0	$8	$395	$0	$52	$3060	$279	$0	$0	$1	$284
2001	$8800	$0	$5070	$0	$0	$53	$84	$0	$68	$3130	$138	$0	$0	$2	$252
2002	$11,800	$0	$6730	$0	$0	$4	$141	$0	$77	$4540	$154	$0	$0	$8	$137
2003	$10,900	$0	$5330	$0	$0	$3	$1110	$0	$305	$2420	$1170	$0	$0	$2	$359
2004	$11,300	$0	$7250	$0	$0	$15	$494	$0	$528	$2270	$495	$0	$0	$1	$254
2005	$15,900	$1	$8330	$0	$0	$2	$1380	$0	$685	$4380	$489	$0	$0	$2	$372
2006	$12,900	$2	$7090	$1	$0	$2	$924	$0	$508	$2500	$1400	$0	$0	$18	$477
2007	$14,900	$3	$8050	$1	$0	$1	$2330	$0	$439	$2840	$254	$0	$0	$0	$775
2008	$19,600	$2	$9840	$1	$0	$22	$3440	$0	$646	$4200	$689	$0	$0	$0	$801
2009	$17,700	$2	$7780	$1	$0	$28	$3180	$0	$1570	$4030	$530	$0	$1	$0	$596

Foreign aid for child health for all regions, in millions of 2009 US$. Foreign aid was only searched in continents in which the diseases were among the ten most burdensome. As such, this table does not accurately reflect worldwide aid for all diseases, as not all diseases were searched in all continents. For instance, fire was among the ten most burdensome diseases only in Sub-Saharan Africa and Europe, so the total listed for fire only reflects the total for those two regions

Overall, the number of DALY varied widely by region (Table 2). Asia and sub-Saharan Africa each accounted for over 350 million DALY in 2000, while Americas, Europe, and Middle East and North Africa had between 15 and 30 million DALY. In every region, the total number of DALY decreased between 2000 and 2012, the 2 years for which we have data. Each region had a reduction in DALY of at least 25%, and Americas, Asia, and Europe neared a 50% reduction.

**Table 2:** Disability Adjusted Life Years by Disease and Region

Americas			Asia			Europe			Middle East and NorthAfrica			Sub-Saharan Africa		
	2000	2012		2000	2012		2000	2012		2000	2012		2000	2012
Newborn	15,349	9075	Newborn	176,562	118,761	Newborn	7.929	5079	Newborn	12,993	9971	Vacdne	110,979	67,060
Vacdne	5.909	2690	Vaccine	119,870	49,281	Vacdne	3934	1678	Vaccine	6341	3239	Newborn	85.950	91,532
Diarrhea	3394	1225	Diarrhea	59,857	24,971	Diarrhea	1524	719	Diarrhea	3141	1508	Malaria	67,641	43,049
Anemia	1262	1052	Anemia	8405	8129	Anemia	534	555	Anemia	638	723	Diarrhea	50,102	31,888
Malnutrition	994	353	Malnutrition	10,677	4373	Endocrine	236	184	Road	513	434	HIV	17,740	8472
Endocrine	385	336	Drowning	6771	3639	Malnutrition	206	105	Malnutrition	566	324	Malnutrition	10,994	10,913
Road	319	235	Syphilis	5193	2090	Fire	166	104	Endocrine	365	358	Tuberculosis	5330	4280
Drowning	336	200	Road	3112	1961	Road	140	107	Syphilis	223	154	Anemia	3731	4891
Syphilis	304	154				Leukemia	122	99	Drowning	209	153	Syphilis	4483	3.739
HIV	362	90				Drowning	257	172				Fire	3274	3913
Total	28,615	15,409	Total	390,447	213,204	Total	15,047	8801	Total	24,987	16,863	Total	360,224	269,736

Disability adjusted life years by disease and by region, in thousands, for years 2000 and 2012

Table 2. Disability adjusted life years by disease and by region, in thousands, for years 2000 and 2012.

Aid was analyzed in absolute terms and by aid per DALY for each continent to examine for patterns (Additional file 1, Figs 1a-e and 2a-e). The figures reveal several patterns. Except for in sub-Saharan Africa, aid was primarily directed at malnutrition and diarrhea. Further, the overall amount of aid to Americas, Asia, Europe, and Middle East and North Africa, was stable over our study period. However, the amount of aid per DALY increased in each of these regions over our study period, due to decreased DALY. In contrast, CHA for sub-Saharan Africa increased dramatically over our study period, from $1.6 billion in 1996 to $10 billion in 2009. While much CHA in sub-Saharan Africa was directed at malnutrition and diarrhea, as was the case with the other regions, HIV and malaria are also major recipients of CHA in this region. CHA for almost all diseases in sub-Saharan Africa increased over our study period, including vaccine preventable diseases ($32 million in 1996 to $320 million in 2009), newborn diseases ($19 million to $270 million), malaria ($6.2 million to $1.6 billion), HIV ($230,000 to $3.1 billion), and diarrheal diseases ($1.1 billion to $2.4 billion). There was a corresponding increase in CHA per DALY in sub-Saharan Africa: CHA per DALY increased from $0.09 in 1996 to $36.47 in 2009 for Malaria, and from $0.01 to $361.19 for HIV.

**Fig. 1:**
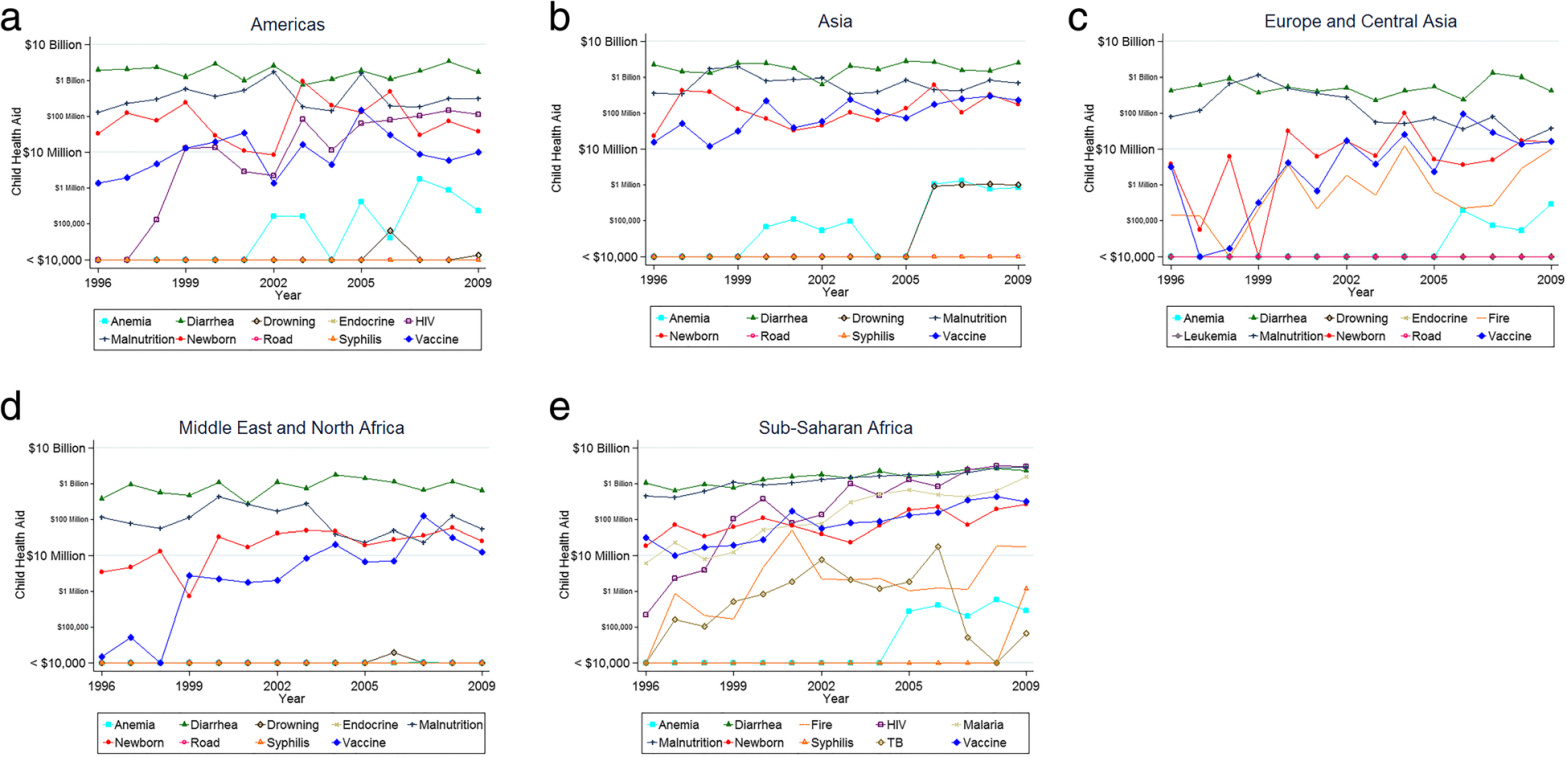
**a** Child health aid by disease: Americas. **b** Child health aid by disease: Asia. **c** Child health aid by disease: Europe. **d** Child health aid by disease: Middle East. **e** Child health aid by disease: Sub-Saharan Africa. Shows the amount of child health aid targeted at specific diseases from 1996 to 2009, for each region studied. A logarithmic scale is used to better visualize the trend, which shows large sums of health aid being targeted towards the highest-receiving diseases, and relatively small amounts of health aid targeted towards other diseases

**Fig. 2:**
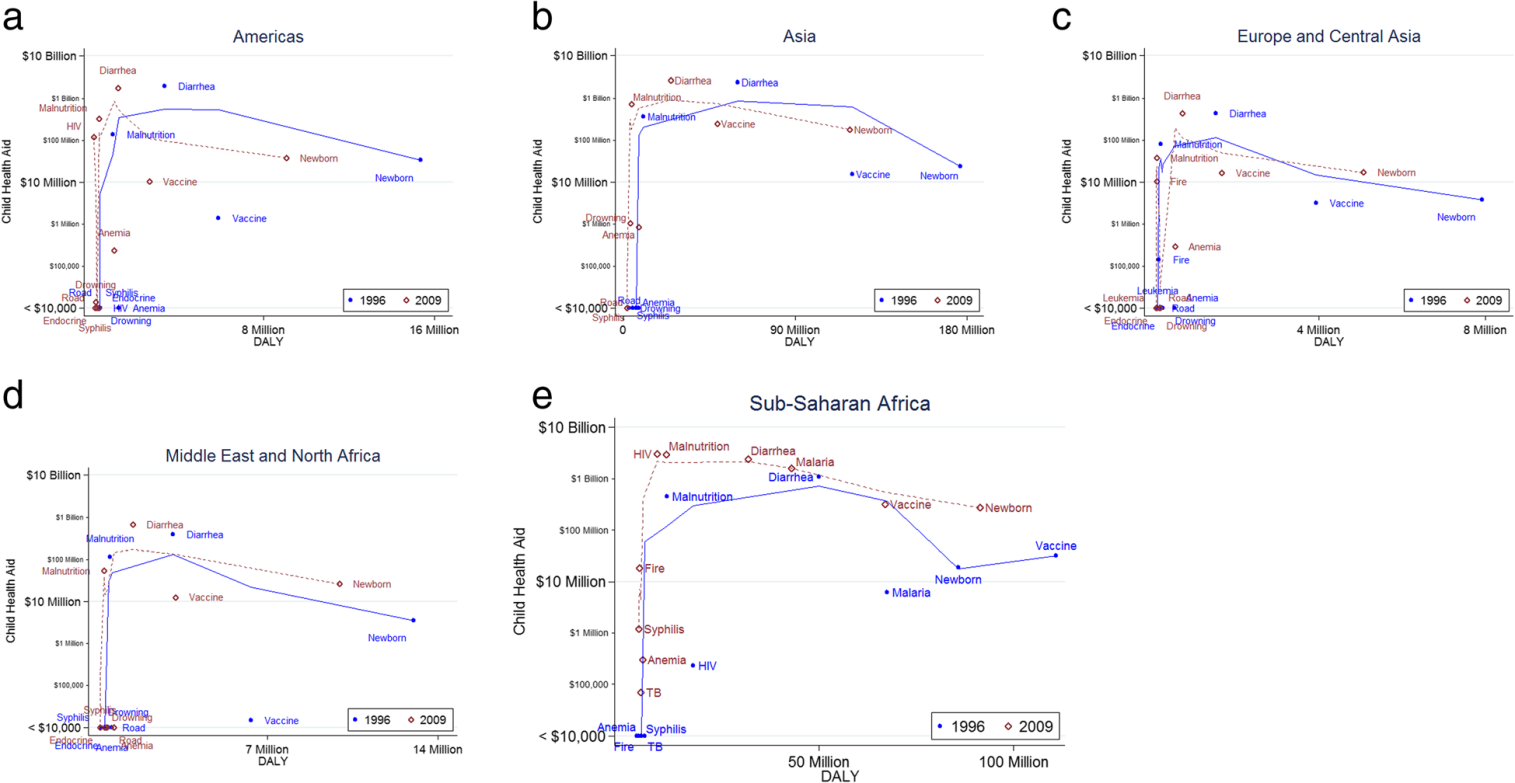
**a** Child health aid and disability adjusted life years: Americas. **b** Child health aid and disability adjusted life years: Asia. **c** Child health aid and disability adjusted life years: Europe and Central Asia. **d** Child health aid and disability adjusted life years: Middle East and North Africa. **e** Child health aid and disability adjusted life years: Sub-Saharan Africa. DALY = Disability adjusted life year. Child health aid and DALY for 1996 and 2009, the first and last year of our study period, are presented in scatterplots for each region. A narrow bandwidth (0.6) lowess curve is used to fit the trends, which consistently show a quick rise in child health aid as DALYs increase from the least burdensome diseases to the moderately burdensome, followed by a stabilization or plateau of the amount of child health aid as DALYs increase towards the most burdensome diseases. The trends for 1996 and 2009 are similar, and there is less burden and more child health aid in 2009 compared to 1996

In all regions, the burden associated with newborn illness was the 1st or 2nd highest (Table 1). Below that, the rank of most burdensome diseases varied by region. The Americas, Asia, Europe and Central Asia, and Middle-East and North Africa have similar lists of burdensome diseases. Vaccine-preventable diseases, diarrhea, and iron-deficiency anemia ranked as the 2nd, 3rd, and 4th most burdensome diseases in each of these continents. Syphilis, malnutrition, and endocrine and blood disorders were often also among the ten most burdensome in these continents, along with conditions of physical injury such as drowning and road accidents. In contrast to the other 4 regions, the most burdensome diseases in Sub-Saharan Africa included more infectious diseases such as malaria, HIV, and tuberculosis.

The relationship between a disease’s burden and the amount of CHA focused on it was examined by plotting these values (Fig. 2a-e). In all continents, diarrheal diseases and malnutrition receive the most CHA though they do not have the highest burden. In the graphical representation, these diseases consistently appear in the top left corner, signifying high CHA and relatively low burden. In contrast, newborn diseases and vaccine-preventable diseases are responsible for the most burden, and receive less aid than diarrheal diseases and malnutrition. Notably, in Sub-Saharan Africa, HIV and malaria became highly funded during our study period, with HIV ranking as the highest funded disease in the region in 2009 (Fig. 2e). Overall, there is no clear pattern between burden and CHA. The lowess curves fit to each set of data (Fig. 2a-ep) show a consistent pattern: the amount of CHA rises quickly as DALYs increase from lower burden to moderate burden, then CHA stabilizes as DALYs increase from diseases of moderate burden to those with the highest burden.

There was a large difference in CHA per DALY between different regions. Overall, Asia received the least CHA per DALY, at less than $20 for each year studied. CHA per DALY increased from $4 to $39 over our study period in sub-Saharan Africa, and was near or above $100 throughout the study period in the Americas. This large difference is primarily due to certain diseases targeted with high CHA. For instance, diarrheal diseases were targeted with over $1000 per DALY in the Americas at the end of our study period, while in Asia diarrheal diseases received around $100 per DALY. For vaccine-preventable diseases, CHA per DALY is similar between regions. However, for newborn diseases, Asia received relatively little CHA compared to other regions. In 2009, for example, CHA for newborn diseases was $1.50 per DALY in Asia, compared to $4.21 (Americas), $3.31 (Europe), $2.61 (Middle East and North Africa), and $4.74 (sub-Saharan Africa).

## Discussion

In this study, we have shown many large disparities in CHA worldwide. Our data reveal that funding for different childhood diseases varies widely; conditions of physical injury and complex medical conditions receive much less CHA than malnutrition, diarrheal diseases, and HIV relative to burden of disease. Further, our findings indicate that the five world regions receive hugely different amounts of CHA. Taken together, our findings suggest that allocation of CHA does not follow the global distribution of childhood burden of disease.

We found that some diseases cause relatively little burden and receive the most CHA, including malnutrition, diarrhea, and HIV. These diseases consistently receive more CHA then the most burdensome disease categories: vaccine-preventable diseases and newborn disease. Lack of clear relationship between burden and health aid was previously described in a report of overall health aid, which found that HIV, malaria, and water and sanitation were relatively highly funded, and that child health and non-communicable diseases were relatively under-funded [4]. However, it is important to note that the true effect of malnutrition may be hard to estimate because it is both a primary cause of morbidity and mortality and also a risk factor for exacerbating the health consequences of other disease states. As such, analyzing malnutrition along with other primary causes of morbidity according to its cause-specific morbidity likely underestimates its actual effect, and so distorts the relationship between burden and CHA.

Our results also echo previously described growth in overall health aid, which has found a 5–10% increase in funding annually from 1990 to 2010 [1]. Worldwide, we found that CHA increased from $7.4 billion in 1996 to $17.7 billion in 2009 for our study diseases, a 6.4% annual growth rate over the 14 years. However, the majority of this growth was due to CHA for Sub-Saharan Africa, which increased from $1.6 billion in 1996 to $10 billion in 2009, a 14% annual increase. In contrast, CHA for Europe actually decreased slightly, and CHA for Americas increased only marginally.

Although the growth trend is similar between our study and others, the total amount of CHA is significantly higher in our study. For instance, total aid for maternal and child health was previously reported as nearly $4 billion in 1996 [1] (in 2014 dollar terms), whereas we found $7.4 billion in 1996. Multiple factors account for our methodology reporting more CHA. First, we included CHA for more diseases in our definition of child and maternal health, including malaria, which has a heavy burden on children, as well as HIV and tuberculosis, if the project description mentioned children. Further, our search algorithm included terms that are not commonly included in health aid. Drinking water and food aid accounted for a large percent of the CHA in our analysis for diarrheal diseases and malnutrition, respectively. Although these are not medical interventions, we believe it is appropriate to include these terms in our analysis of CHA.

Commonly, an intended goal of foreign aid is to assist the recipient’s health system performance. For CHA, this means to treat and prevent as much disease as possible. While this logic might suggest that health aid should follow the burden of disease, it must be recognized that there are several other factors to consider, including the different cost-effectiveness of interventions for different diseases [10], the wealth and capacity of the recipient nations, and the disparate priorities and capabilities of major donors.

Reports of the cost-effectiveness of treatment and prevention of disease in low- and middle-income countries show large disparities. While not focused on CHA, Laxminarayan’s analysis described interventions according to cost per DALY averted, and found several to be cost-effective therapies, such as immunizations, anti-retroviral treatment to prevent mother-to-child transmission of HIV, and management of childhood lower respiratory infections [10]. On the other hand, several therapies were described as not cost effective, including treatment for cardiovascular disease, mental health, isoniazid treatment of TB, and seatbelt laws and random driver breathalyzer tests for road safety. Prioritizing cost-effectiveness, rather than only burden, in health aid disbursement has been suggested as one method to maximize the impact of aid [10, 11]. Previous regression analysis suggests that health aid is positively correlated with cost-effectiveness; that is, more aid is given to diseases with more cost-effective interventions [11]. However, the extent to which that relationship implies informed decision-making by donors is unclear.

Additionally, donors have priorities and strengths that affect their decisions. Donor priority has been reported as at least as important as recipient need, and there is also evidence of an effect due to colonial history and political alliances [12]. Further, donors fund initiatives they believe they are uniquely suited for. For instance, the World Bank addresses long-term infrastructure development, while other donors focus on drug delivery, such as the United States government [4]. Thus, there are factors other than disease burden to consider when determining the most efficacious distribution of CHA.

Further, recipient-country income is likely another factor that can explain why CHA does not correlate more closely to burden of disease. Though outside the scope of this analysis, it is likely that some donors use recipient country income as a factor in making decisions. This could be why Asia, with many middle-income countries, received less CHA per DALY than predominately low-income sub-Saharan Africa, while the two regions had comparable burden. It is important for future work to investigate this trend further.

Alongside the difficulty in determining the optimal distribution of CHA, there is significant difficulty in accurately determining CHA commitment amounts. As previously stated, most information regarding health aid does not allow for granular analysis of CHA, which has limited such analysis previously. IHME, World Bank, and OECD all report foreign aid amounts, but group childhood diseases into one disease category.

While AidData has allowed for more granular analysis due to its text description of each project, it also has clear limitations. As previously stated, small private foundations and NGOs are not included in AidData. As such, our analysis is likely not accounting for about 15% of CHA, the amount of total health aid that comes from small donors and NGOs [1]. Further, it is possible that these smaller groups have different funding priorities than the donors included in AidData, which would impact the patterns of funding described in this analysis.

Further, there are forms of international assistance that are not considered foreign aid, and so are not included in databases of foreign aid. For instance, although our analysis described no funding for road safety, there are campaigns to increase road safety by lobbying for seatbelt and child-seat law, such as that by Bloomberg Philanthropies [13]. However, these were not captured by AidData, and so were not included in our analysis.

Also, there were foreign aid projects in AidData focused on repairing roads, but none specified that the intention was to increase safety for children. Of course, newly renovated roads would likely be safer, and so one alternate approach to our analysis was to include all aid focused on a topic, regardless of whether it included specifications for child health. However, the focus of this analysis is aid for children’s health, and we believe our methodology resulted in a child-specific analysis.

There are also major limitations to tracking aid in general. A report by the Center for Global Development highlighted the difficulties due to multiple donors and lack of standardized data [14]. In addition, our algorithm is not perfect. In more precise language, our algorithm can be described by its sensitivity, which describes what percent of the projects which truly focus on a disease the algorithm successfully found, and its specificity, which describes the percent of projects not related to a disease that the algorithm successfully excluded. For example, in our search for projects related for fires, the word “firearm” was flagged by our algorithm searching for “fire,” which would have been a false positive result, leading to a lower specificity. In this case, we added an exclusion for “firearm,” as well as “ceasefire” and thoroughly read through all results of our search in order to maximize the algorithm’s sensitivity and specificity. Based on thorough testing and samples run between the algorithm and a human reader, we estimate that our sensitivity and specificity are both close to 95%.

Another specific limitation of our analysis was that immunization funding often did not specify which immunization, and so we grouped all vaccine-related diseases into one category. While we feel this is appropriate due to the similarity in preventive strategy for this group of diseases, such grouping limits the exactness of our analysis. We were further limited by the disease category of lower respiratory tract infection, which is a broad category, with some vaccine-preventable components, such as pneumonia and influenza, and some non-vaccine related diseases, such as other bacterial pneumonias. Future work should aim to break this disease group into specific diseases.

## Conclusions

Our analysis has highlighted several patterns of CHA, such as the lack of correlation between a disease’s burden and the amount of CHA targeted towards it. Further, we discussed the disparate amount of funding received per DALY by different continents, with Asia receiving relatively little and Sub-Saharan Africa receiving relatively large amounts of CHA per DALY. Given the available data, we believe this is the most thorough and accurate description of CHA possible at this time. Further work is needed to better describe CHA distribution and to evaluate the optimal distribution of CHA. This and future work will assist policy-makers to realize current trends and to assess for more optimal patterns.

## Additional file

Additional file 1: Appendix. (DOCX 1230 kb)

## Abbreviations

CHA: Child-specific health assistance
DALY: Disability adjusted life year
GFATM: Global fund to fight AIDS, Tuberculosis, and Malaria
IHME: Institute for Health Metrics and Evaluation
NGO: Non-governmental organization
OECD: Organization of Economic Cooperation and Development
WHO: World Health Organization
