# Supplementary material for: The relationship between burden of childhood disease and foreign aid for child health
Source: BMC Health Serv Res. 2017 Sep 15;17:655. doi: 10.1186/s12913-017-2540-5 (PMC5602827; doi:10.1186/s12913-017-2540-5)
Supplement: Additional file 1: — Appendix. (DOCX 1230 kb) [file 12913_2017_2540_MOESM1_ESM.docx]

S1 Appendix Table 1. List of Low and Middle Income Countries Included in Analysis

Americas: Argentina, Barbados, Belize, Bolivia, Brazil, Chile, Colombia, Costa Rica, Cuba, Dominican Republic, Ecuador, El Salvador, Guatemala, Guyana, Haiti, Honduras, Jamaica, Mexico, Nicaragua, Panama, Paraguay, Peru, Suriname, Trinidad and Tobago, Uruguay, Venezuela

Asia: Afghanistan, Bangladesh, Bhutan, Cambodia, China, Fiji, India, Indonesia, Laos, Malaysia, Maldives, Mongolia, Myanmar, Nepal, North Korea, Pakistan, Papua New Guinea, Philippines, Solomon Islands, South Korea, Sri Lanka, Thailand, Vietnam

Europe and Central Asia: Albania, Armenia, Azerbaijan, Belarus, Bosnia and Herzegovina, Bulgaria, Croatia, Czech Republic, Estonia, Georgia, Hungary, Kazakhstan, Kyrgyzstan, Latvia, Lithuania, Macedonia, Moldova, Montenegro, Poland, Romania, Russia, Serbia, Slovakia, Tajikistan, Turkey, Turkmenistan, Ukraine, Uzbekistan

Middle East and North Africa: Algeria, Bahrain, Djibouti, Egypt, Iran, Iraq, Jordan, Lebanon, Libya, Malta, Morocco, Oman, Saudi Arabia, Syria, Tunisia, Yemen

Sub-Saharan Africa: Angola, Benin, Botswana, Burkina Faso, Burundi, Cameroon, Cape Verde, Central African Republic, Chad, Comoros, Congo, Cote d'Ivoire, Democratic Republic of the Congo, Equatorial Guinea, Eritrea, Ethiopia, Gabon, Gambia, Ghana, Guinea, Guinea Bissau, Kenya, Lesotho, Liberia, Madagascar, Malawi, Mali, Mauritania, Mauritius, Mozambique, Namibia, Niger, Nigeria, Rwanda, Senegal, Sierra Leone, Somalia, South Africa, Sudan, Swaziland, Tanzania, Togo, Uganda, Zambia, Zimbabwe

S1 Appendix Table 2. Search Algorithm

Anemia: Anemia/Anaemia, iron fortified, iron containing

Diarrhea: diarrhea, balantidium, giardia, cryptosporidium, lambliasis, rotavirus, Norwalk, oral rehydration therapy, electrolyte, gastroenteritis, cholera, zinc, vitamin A, carotene, retinol, typhoid, salmonella, dysentery, shigella, e. coli, campylobacter, Yersinia enterocolitica, colitis, amebiasis, drinking water, sanitation, potable

Drowning: drown

Endocrine Blood Disorders: favism, glucose 6 phosphate dehydrogenase, hexokinase, pyruvate kinase, thalassemia, sickle cell, spherocytosis, elliptocytosis, hemoglobinopathy, hemolytic anemia, paroxysmal nocturnal hemoglobinuria, red cell aplasia, aplastic anemia, sideroblastic anemia, disseminated intravascular coagulation, factor viii deficiency, factor ix deficiency, hemophilia, von willebrand, thrombophilia, bernard soulier, glanzmann thrombocytopenia, idiopathic thrombocytopenic purpura, thrombocytopenia, agranulocytosis, neutropenia, eosinophilia, hyposplenism, hypersplenism, methemoglobinemia, erythrocytosis, sarcoidosis, hypogammaglobulinemia, hypergammaglobulinemia, severe combined immunodeficiency syndrome, wiskott aldrich, digeorge, common variable immunodeficiency, cryoglobulinemia, myxedema, thyrotoxicosis, thyroiditis, hypoparathyroid, hyperparathyroid, pituitary, hypopituitary, acromegaly, hyperprolactinemia, syndrome of inappropriate anti-diuretic hormone, cushing syndrome, adrenogenital, hyperaldosteronism, polycystic ovarian syndrome, 5 alpha reductase, precocious puberty, thymus, carcinoid, obesity, phenylketonuria, alkaptonuria, albinism, maple syrup urine, lactose intolerance, lactase deficiency, glycogen storage, fructosuria, galactokinase, galactosemia, sphingolipid, sandhoff, tay sach, mucopolysaccharidosis, hypercholesterolemia, lipoprotein deficiency, lesch nyhan, porphyria, gilbert syndrome, crigler najjar, hemochromatosis, amyloidosis, cystic fibrosis

Fire: fire, burn.
Excluded from fire: firearm, ceasefire

HIV: HIV, AIDS

Leukemia: leukemia

Malaria: malaria, bednet, insecticide

Newborn: newborn, neonatal, peripartum, low birth weight, obstetric, maternity, truncus arteriosis, congenital, ventricular septal defect, atrial septal defect, pulmonary valve atresia, ebstein anomaly, hypoplastic left heart, dextrocardia, patent ductus arteriosis, aortic coarctation, aortic atresia, aortic stenosis, total anomalous venous return, anencephaly, spina bifida, meningocele, myelocele, folate, vitamin b12

Protein Energy Malnutrition: kwashiorkor, malnutrition, nutrition, protein, marasmus, emaciation, stunting, food aid

Road: (vehicle, automobile, car, bus, traffic) + (accident, collision), road­­, seatbelt, childseat

Syphilis: syphilis, treponema, pallidum

Tuberculosis: tuberculosis, tuberculoma

Vaccine Preventable: vaccine, immunization, pneumonia, haemophilus, HIB vaccine, PRP vaccine, respiratory infection, influenza, respiratory syncytial virus, pneumococcal, pentavalent, measles, MMR, meningitis, meningococcal, cerebrospinal, TDAP, DPT/DTP, pertussis, tetanus, bordatella, whooping, trismus, lockjaw

S1 Appendix Table 3

**
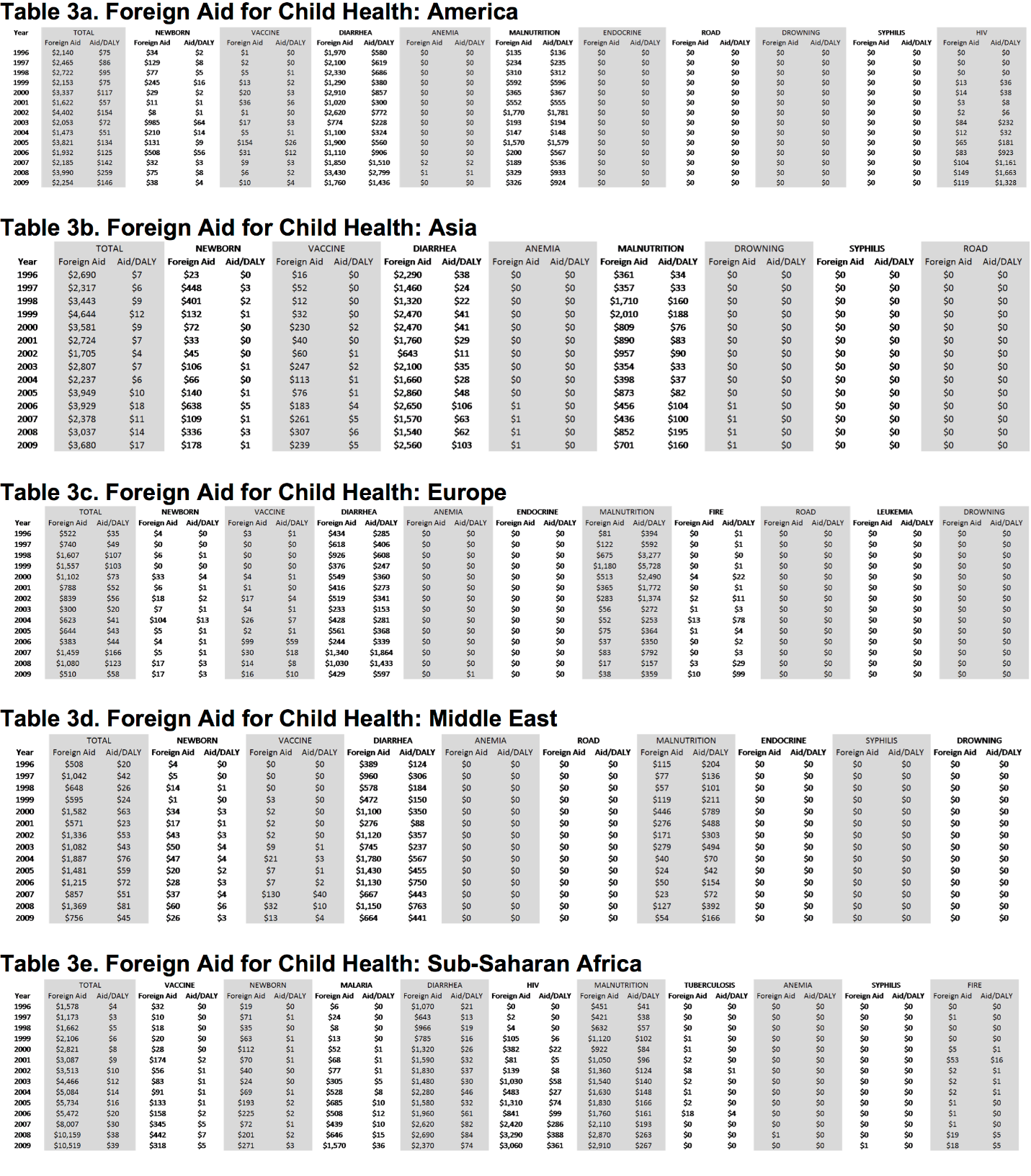
**

**Table 3.** Total foreign aid for child health, in millions of US$, and foreign aid per disability adjusted life year (DALY), in US$ per DALY, presented for each region.
